# Atherogenic Risk Assessment among Persons Living in Rural Uganda

**DOI:** 10.1155/2016/7073894

**Published:** 2016-06-22

**Authors:** Clara Wekesa, Gershim Asiki, Ivan Kasamba, Laban Waswa, Steven J. Reynolds, Rebecca N. Nsubuga, Rob Newton, Anatoli Kamali

**Affiliations:** ^1^Medical Research Council/Uganda Virus Research Institute Uganda Research Unit on AIDS, Entebbe, Uganda; ^2^Division of Intramural Research, National Institute of Allergy and Infectious Diseases, National Institutes of Health, Bethesda, MD 20892, USA; ^3^Johns Hopkins School of Medicine, Baltimore, MD 21205, USA

## Abstract

*Background*. Hypertension and dyslipidemia are independent risk factors for coronary heart disease and commonly coexist. Cardiovascular risk can be reliably predicted using lipid ratios such as the atherogenic index, a useful prognostic parameter for guiding timely interventions.* Objective*. We assessed the cardiovascular risk profile based on the atherogenic index of residents within a rural Ugandan cohort.* Methods*. In 2011, a population based survey was conducted among 7507 participants. Sociodemographic characteristics, physical measurements (blood pressure, weight, height, and waist and hip circumference), and blood sampling for nonfasting lipid profile were collected for each participant. Atherogenic risk profile, defined as logarithm base ten of (triglyceride divided by high density lipoprotein cholesterol), was categorised as low risk (<0.1), intermediate risk (0.1–0.24), and high risk (>0.24).* Results*. Fifty-five percent of participants were female and the mean age was 49.9 years (SD ± 20.2). Forty-two percent of participants had high and intermediate atherogenic risk. Persons with hypertension, untreated HIV infection, abnormal glycaemia, and obesity and living in less urbanised villages were more at risk.* Conclusion*. A significant proportion of persons in this rural population are at risk of atherosclerosis. Key identified populations at risk should be considered for future intervention against cardiovascular related morbidity and mortality. The study however used parameters from unfasted samples that may have a bearing on observed results.

## 1. Background

Globally, cardiovascular disease (CVD) is the leading cause of death with up to 80% occurring in low and middle income countries (LMICs) [1]. Premature death due to CVD is rapidly growing in Sub-Saharan Africa (SSA) [2]. Hypertension and dyslipidemia are thought to be the major risk factors for CVD [3]. A systematic review on hypertension in SSA yielded 17 studies conducted between 1998 and 2008 in 11 countries and found an overall prevalence of 16.2%, with small variation between urban and rural communities [4]. In Uganda, the prevalence of hypertension ranges from 14.6% in the rural areas [5] to 27.2% in the periurban area [6]. In Western populations, dyslipidemia among patients with hypertension is a common finding. The prevalence of dyslipidemia coexisting with hypertension has been estimated at 15–24% [3, 7]. Over 40% of all newly diagnosed hypertensive patients have at least one lipid abnormality [8]. There is dearth of data on the burden of dyslipidemia among patients with hypertension in SSA.

Hypertension and dyslipidemia are both independent modifiable risk factors for CVD. However, some studies suggest that dyslipidemia may play a role in the development of hypertension via endothelial damage [9]. The risk for CVD is higher in the presence of both hypertension and dyslipidemia than by the individual disease entities [10]. In high income countries, strategies targeting the reduction of serum cholesterol levels provide the single most important benefit against CVD resulting from coronary heart disease (CHD) when evaluated in a cohort setting [11]. A review on a number of cohort studies and randomised clinical trials found that a 10% decrease in cholesterol reduced the risk of CHD by 50% at the age of 40 and this benefit reduces to 20% by the age of 70 [12]. In contrast, blood pressure control provides only a 20% risk reduction of mortality due to CHD in high income settings.

Current medical practice encourages risk stratification for all persons at risk of CHD including those with hypertension. This is advantageous for timely intervention in preventing CHD and its sequelae. Cardiovascular risk assessment based solely on serum low density lipoprotein cholesterol (LDL-C) has been found to be inadequate [13, 14] and even in persons on statin therapy with normal or near normal LDL-C serum levels, there remains risk for future cardiovascular events. Hypertriglyceridemia has been identified as an independent cardiovascular risk factor [15, 16]. Hypertriglyceridemia is a proxy for atherogenic dyslipidemia (elevated TG (triglyceride) and low high density lipoprotein cholesterol) [16, 17], a component of the metabolic syndrome and a known risk factor for CHD. Despite interaction with other lipids that might increase atherogenic risk and in-subject variation, elevated TG has been shown to be a good predictor of CHD in consideration of the high density lipoprotein cholesterol (HDL-C) levels [17] and is used in clinical practice to predict atherosclerosis [17, 18]. The lipid ratio log [TG/HDL-C] (atherogenic index) provides an alternative simple option for risk stratification. The use of this ratio is to reflect the balance between risk and protective lipoprotein forces [15] as well as acting as a correlate with LDL-C particle size [19], LDL-phenotype B, and small HDL-C particles [16]. This ratio has also been evaluated as a prognostic tool in predicting CHD [16] and its predictive value far outweighs that of absolute lipid parameters [20, 21] as well as the TC (total cholesterol)/HDL ratio [21] given its strong correlation with lipoprotein particle size.

Uganda bears a significant burden of hypertension [22] and like other SSA countries is not presently equipped with the necessary resources, expertise, and technology to manage the CVD sequelae. A recent study done within a rural population reflected the dominant dyslipidemic pattern to be that of low serum HDL-C levels and a good proportion with hypertriglyceridemia [23]. Whereas there are well-recognised recommendations on the use of serum LDL-C and TC for cardiovascular risk assessment, there is limited progress in addressing concerns with low serum HDL-C which may lead to underestimation of CHD risk [24]. The atherogenic index provides a simple and reliable method for point of care risk assessment that can be done by nonspecialists even within hard-to-reach areas. The aim of this study was to assess the atherogenic risk profile using the atherogenic index among people living in rural Uganda.

## 2. Methods

### 2.1. Design and Setting

A population based survey was conducted in 2011 among 7507 individuals aged 13 years and above living in an established rural cohort in southwestern Uganda, the General Population Cohort (GPC) consisting of 25 villages. This cohort has been in existence for the last 25 years and was initially established for the purpose of investigating HIV trends in rural Uganda. Research activities have since been broadened to include noncommunicable diseases, assessing burden and risk factors [25].

### 2.2. Study Procedures

#### 2.2.1. Questionnaire

Sociodemographic information was collected for each participant and entered into an electronic questionnaire. This information included age, sex, and occupation, level of education, marital status, smoking history, and use of alcohol. The level of urbanicity was determined and presented as described elsewhere [26].

#### 2.2.2. Physical Measurements

Blood pressure measurement, height, weight, and hip and waist circumferences were measured as described by Riha et al. 2014 [26]. Participants found to have systolic blood pressure ≥140 mmHg and/or diastolic pressure ≥90 mmHg were referred to as having hypertension. Participant categorisation using body mass index (BMI) was as follows: BMI ≤ 18.5, “underweight,” BMI 18.5–24.9, “normal weight,” BMI 25–29.9, “overweight,” and BMI ≥ 30, “obese”. Categorisation by waist-hip ratio (WHR) was as follows: abnormal in male if >0.95 and abnormal in females if >0.8.

#### 2.2.3. Laboratory Measurements

Participants underwent phlebotomy for purposes of a nonfasting lipid profile (TC, TG, HDL-C, and low density lipoprotein cholesterol (LDL-C)), HIV serology, and glycated haemoglobin (HbA1c). All blood samples were transported on the same day to a central laboratory for sample processing and testing. Lipid profile analysis was done using the enzymatic colorimetric assay by the Cobas Integra 400 Plus. Cut-off values for the different lipid parameters representative of dyslipidemia were as follows: TC > 5.2 mmol/L, TG > 2.3 mmol/L, LDL-C > 3.4 mmol/L, and HDL-C < 1.04 mmol/L, male, and <1.3 mmol/L, female. Participants with HbA1c ≥ 6.5% were termed as diabetic, HbA1c 6–6.49% were termed as high risk for diabetes, and HbA1c < 6% were termed as low risk for diabetes [27]. HIV testing was done in accordance with the Ministry of Health guidelines [28].

### 2.3. Data Analysis

To assess the atherogenic risk we used the atherogenic index defined as log(triglycerides/HDL-C). The risk was categorised into low (<0.1), intermediate (0.1–0.24), and high (>0.24) [21].

The sample was described using proportions by sex. Age and systolic and diastolic blood pressure were summarized with means and corresponding standard deviations (SD). Atherogenic risk was determined using proportions in each category and the chi-squared test was used to compare proportions. We used the ordered logistic regression model to evaluate the effect of demographic characteristics, blood pressure, lipid profiles, and HIV status on atherogenic risk and the results were presented by sex. To avoid residual confounding and loss of power, continuous lipid measures were modelled as continuous rather than categorised. The continuous variables were included into the model as centered and assessed for nonlinearity by fitting a first-order fractional polynomial to the data. The proportional odds assumption in using the ordered logistic regression was tested. Analysis was done using STATA 13 (Stata Corp, College Station, TX).

### 2.4. Ethical Consideration

The study was conducted in accordance with the principles of the Declaration of Helsinki and was approved by the Uganda Virus Research Institute Research Ethics Committee (UVRI REC) in Entebbe, Uganda, and by the Uganda National Council of Science and Technology (UNSCT). Written informed consent was obtained from all study participants. For participants below the age of 18 years, parental consent and formal assent to participate in the study were obtained as per the Ugandan National Guidelines for Research Involving Humans as Research Participants [29]. Participants with hypertension and raised serum lipids were referred to a care clinic for further management and received standard of care (health education on risk factor prevention and/or control and drug therapy with statins).

## 3. Results

### 3.1. Baseline Characteristics

The study enrolled 7507 participants, the majority were females (55%), and the mean age was 49.9 years (SD ± 20.2). There were equal distributions of participants across the different levels of urbanicity, most of the participants were under the age of 30, and the use of alcohol and tobacco was more common among the male participants (Table 1).

From the physical and laboratory measurements we found more female participants to be overweight and obese and have an abnormal WHR. Approximately 18% of participants were identified as having hypertension and only 2% had controlled hypertension on treatment. The most common form of dyslipidemia was low serum HDL-C levels (Table 1).

### 3.2. Atherogenic Risk

**Figure 1:**
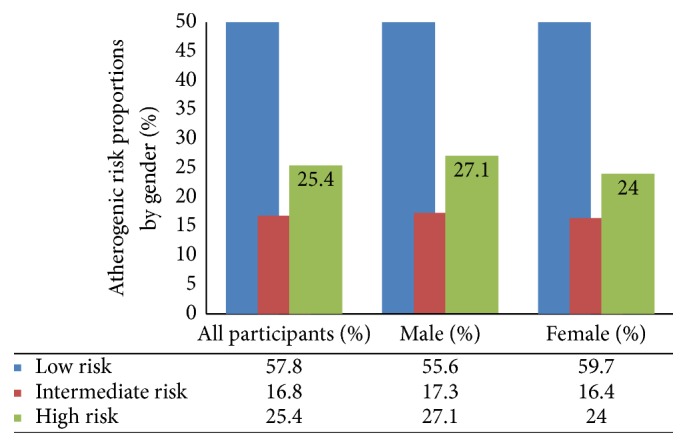
Atherogenic risk profile among study participants stratified by gender.

The proportion of participants with intermediate and high atherogenic risk was 17% and 25%, respectively, with the greater proportion at high risk being male participants (Figure 1).

### 3.3. Risk Factors for High Atherogenic Risk

Participants with hypertension (particularly those with suboptimal control on treatment), residents from less urbanised villages, HIV-infected participants not on antiretroviral therapy (ART), overweight and obese participants, and participants with abnormal HbA1c had significantly higher odds of having high atherogenic risk (Table 2).

Being female, 30 years of age and above, having attained higher level of education, current consumption of alcohol, and being on ART were significantly associated with lower odds of having high atherogenic risk. Abnormal total cholesterol serum levels and history of smoking had no association with atherogenic risk.

## 4. Discussion

With increasing globalisation, varying degrees of urbanisation exist even within rural areas [26]. Urbanisation influences environmental and individual factors that may have impact on cardiovascular risk. In this study we assessed the risk for atherosclerosis using the atherogenic index in a rural Ugandan cohort. The atherogenic index in comparison to absolute lipid parameters and to TC/HDL-C ratio provides more accurate estimates of cardiovascular risk [30] and is more likely to be a better screening tool. Our study is one of the few studies in the region that provides insight into the use of the atherogenic index and having a cohort already in establishment provides the unique opportunity to conduct pilot studies that generate hypotheses to guide future research in this field. This study also assesses the use of the atherogenic index in a widely distributed rural population and is not only restricted to most at-risk populations. The atherogenic index requires minimal expertise and can be widely implemented as a screen method for cardiovascular disease even within remote locations.

A significant proportion of participants were at risk of atherosclerosis. A population based survey done in Iran and using the atherogenic index found a similar high estimate of atherogenic risk [31]. The INTERHEART study, an observational study involving 52 countries spread across all continents, found that persons with history of myocardial infarction had three times the odds of having a raised ApoB/ApoA1 ratio (surrogate for atherogenic dyslipidemia) compared to persons without history of myocardial infarction [32]. An observational study done in Brazil found that persons with coronary disease had two times the odds of having an abnormal TG/HDL-C ratio [33]. In our study participants with hypertension had higher odds of risk for atherosclerosis. We found that 27% of participants with hypertension had a high atherogenic risk, a higher estimate compared to a Nigerian study that found a lower proportion of patients with hypertension at risk for CHD using LDL-C/HDL-C ratios for risk stratification [30]. We attributed this difference in proportions to the atherogenic index being a correlate to lipoprotein size [21] which may predict risk better. Participants on treatment, but with uncontrolled hypertension, were more likely to be at risk of atherosclerosis compared to those not on treatment and those with controlled hypertension. Literature shows that blood pressure control gives minimal reduction of cardiovascular risk especially among those older than 40 years [12] and in our study we found the mean age to be 49 years. This could explain why even among participants on treatment and controlled hypertension atherogenic risk was still high. Participants that were HIV infected and ART naive were more likely to have a high atherogenic risk, a finding similar to other studies done, possibly owing to their high inflammatory state [34]. The use of ART was found to be protective as ART is thought to reduce inflammatory markers and provide cardioprotection [35]. However, other studies have found the use of protease inhibitors (PI) to increase the serum levels of atherogenic lipoproteins [34]. In this population first-line therapy was non-PI based and could further explain our findings. We also observed that a high atherogenic index was less prevalent in participants aged 60 years and above and yet it is more likely that individuals in this age category are more at risk of cardiovascular events. The HIV prevalence in this cohort is higher in the younger age group and we thought this may be the main driver for the increased risk in this age group.

Abnormal HbA1c as a screen for diabetes was also found to be associated with high atherogenic risk. Diabetes affects the integrity of the vasculature and is also associated with other conditions that predispose to CHD, such as hypertension and dyslipidemia that may explain the increased risk. Previous epidemiological studies have shown cholesterol to predict risk of CHD and LDL-C to be associated with future risk [36]; however, we did not find any association between high serum cholesterol levels and atherogenic risk in this rural population. Similar to the Framingham study [36] and the INTERHEART study [32] we demonstrated that obesity and abdominal obesity were associated with increased risk of CHD in this study population. Our study showed that participants from less urbanised villages were more likely to be at high risk for CHD. However previous studies done within this same population showed that persons in less urbanised villages were more likely to indulge in physical activity and eat fruits and vegetables [26], factors thought to reduce risk for CHD. It may be possible that a different set of risk factors, yet to be explored, is responsible for the risk of CHD in this population.

Our study was limited in that we did not derive lipid parameters from fasted samples which may impact on risk estimation if there should be any variation with parameters derived after fasting. We also did not ascertain differences among the subcategories of participants with hypertension that may have accounted for differences in risk profile. The design of the study did not allow making inferences as to casualty. However this study provides insight into estimated cardiovascular risk in a widely distributed rural population taking into account environmental, infectious, and noninfectious risk factors and provides a platform for further research into possible causes of high atherogenic risk whose findings may give information on preventive strategies.

## 5. Conclusion

A significant proportion of persons in this rural Ugandan population are at risk of atherosclerosis. Persons with hypertension, untreated HIV infection, abnormal glycaemia, and obesity appear to be more at risk and may be considered as target groups for intervention programs. Follow-up studies are needed to further assess other risk factors including the role of genetics in cardiovascular risk in this population.

## Figures and Tables

**Table 1:** Sociodemographic, clinical, physical, and biochemical characteristics of cohort by gender.

	All participants	Sex	
	*n* (%)	Male, *n* (%)	Female, *n* (%)
All	7507	3378	4129

*Demographic characteristics*			
*Age, in years*			
13–29	3679 (49.0)	1804 (53.4)	1875 (45.4)
30–59	2978 (39.7)	1226 (36.3)	1752 (42.4)
60+	850 (11.3)	348 (10.3)	502 (12.2)
*Urbanicity score* ^*∗*^			
Quartile 1 (least urban)	1978 (26.4)	884 (26.2)	1094 (26.5)
Quartile 2	1893 (25.2)	848 (25.1)	1045 (25.3)
Quartile 3	1946 (25.9)	888 (26.3)	1058 (25.6)
Quartile 4 (most urban)	1690 (22.5)	758 (22.4)	932 (22.6)
*Highest education level*			
None/up to P4	2112 (28.1)	876 (25.9)	1236 (29.9)
P5–P7	3365 (44.8)	1579 (46.7)	1786 (43.3)
Secondary+	2030 (27.0)	923 (27.3)	1107 (26.8)
*Occupation*			
Subsistence crop	2975 (39.6)	1102 (32.6)	1873 (45.4)
Cash crop cultivator	934 (12.4)	491 (14.5)	443 (10.7)
Noncrop	1043 (13.9)	625 (18.5)	418 (10.1)
Unemployed/full-time education	2555 (34.0)	1160 (34.3)	1395 (33.8)
*Marital status* ^*∗∗*^			
Not married/divorced	3861 (51.5)	1842 (54.6)	2019 (48.9)
Married/living	3638 (48.5)	1532 (45.4)	2106 (51.1)

*Lifestyle characteristics*			
*Smoking*			
Never smoked	6747 (89.9)	2707 (80.1)	4040 (97.8)
Ever smoked daily	760 (10.1)	671 (19.9)	89 (2.2)
*Alcohol consumption*			
Never drinker	4844 (64.5)	2032 (60.2)	2812 (68.1)
Ex-drinker	583 (7.8)	235 (7.0)	348 (8.4)
Current drinker	2080 (27.7)	1111 (32.9)	969 (23.5)

*Biophysical characteristics*			
*BMI group*			
Underweight (<18.5)	1701 (22.7)	1022 (30.3)	679 (16.4)
Normal (18.5–24.9)	4878 (65.0)	2179 (64.5)	2699 (65.4)
Overweight (≥25)	730 (9.7)	156 (4.6)	574 (13.9)
Obese	198 (2.6)	21 (0.6)	177 (4.3)
*Waist-hip ratio (WHR)*			
Normal (≤0.95-m, ≤0.80-f)	4440 (59.1)	3257 (96.4)	1183 (28.7)
Abnormal (>0.95-m, >0.80-f)	3067 (40.9)	121 (3.6)	2946 (71.3)

*Biochemical characteristics*			
*Total cholesterol*			
Desirable (≤5.2 mmol/L)	7084 (94.4)	3270 (96.8)	3814 (92.4)
High (>5.2 mmol/L)	423 (5.6)	108 (3.2)	315 (7.6)
*Triglycerides*			
Desirable (≤2.3 mmol/L)	7190 (95.8)	3261 (96.5)	3929 (95.2)
High (>2.3 mmol/L)	317 (4.2)	117 (3.5)	200 (4.8)
*LDL-C*			
Desirable (≤3.4 mmol/L)	7134 (95.0)	3286 (97.3)	3848 (93.2)
High (>3.4 mmol/L)	373 (5.0)	92 (2.7)	281 (6.8)
*HDL-C*			
Desirable (≥1.04-m, 1.3-f)	1940 (25.8)	1127 (33.4)	813 (19.7)
Undesirable (<1.04-m, 1.3-f), low	5567 (74.2)	2251 (66.6)	3316 (80.3)
*HbA1-C *			
Normal (≤6%)	7469 (99.7)	3359 (99.8)	4110 (99.7)
Raised (≥6.5%)	19 (0.3)	7 (0.2)	12 (0.3)

*Clinical characteristics*			
*Hypertension status*			
Normal BP	6163 (82.1)	2799 (82.9)	3364 (81.5)
Normal BP on treatment	173 (2.3)	56 (1.7)	117 (2.8)
Raised on treatment	119 (1.6)	31 (0.9)	88 (2.1)
Raised BP, not on treatment	1050 (14.0)	492 (14.6)	558 (13.5)
*HIV/ART status*			
Negative	6821 (92.0)	3103 (93.5)	3718 (90.7)
Positive, on ART	201 (2.7)	63 (1.9)	138 (3.4)
Positive, not on ART	396 (5.3)	152 (4.6)	244 (6.0)
*Atherogenic risk*			
Low (<0.1)	4341 (57.8)	1878 (55.6)	2463 (59.7)
Intermediate (0.1–0.24)	1259 (16.8)	583 (17.3)	676 (16.4)
High (>0.24)	1907 (25.4)	917 (27.1)	990 (24.0)

^*∗*^Riha et al. 2014 [26].

^**∗∗**^8 missing pieces of information.

**Table 2:** Factors associated with high atherogenic risk.

Covariates	High atherogenic risk	Crude OR^*ϕ*^		Adjusted OR^*ϕ*^	Overall *p* value
	*n* (%)			b/ci95	
*Hypertension status*					
Normal BP (≤140/90 mmHg) (*n* = 6163)	1538 (25.0)	1	**0.022**	1	**0.007**
Normal BP on treatment (*n* = 173)	53 (30.6)	1.34 (1.01–1.78)		1.32 (0.98–1.79)	
Raised BP (≥140/90 mmHg) on treatment (*n* = 119)	36 (30.3)	1.51 (1.08–2.11)		1.53 (1.07–2.18)	
Raised BP not on treatment (*n* = 1050)	280 (26.7)	1.04 (0.91–1.18)		1.17 (1.00–1.37)	

*Sex*					
Male (*n* = 3378)	917 (27.1)	1	**<0.001**	1	**<0.001**
Female (*n* = 4129)	990 (24.0)	0.85 (0.77–0.93)		0.57 (0.50–0.66)	

*Age, in years*					
13–29 (*n* = 3679)	980 (26.6)	1	**0.001**	1	**<0.001**
30–59 (*n* = 2978)	727 (24.4)	0.88 (0.80–0.97)		0.84 (0.75–0.94)	
60+ (*n* = 850)	200 (23.5)	0.84 (0.71–1.00)		0.71 (0.58–0.87)	

*Urbanicity score*					
Quartile 1 (least urban) (*n* = 1978)	488 (24.7)	1	**<0.001**	1	**0.018**
Quartile 2 (*n* = 1893)	535 (28.3)	1.15 (1.02–1.30)		1.16 (1.02–1.31)	
Quartile 3 (*n* = 1946)	473 (24.3)	0.94 (0.83–1.07)		0.96 (0.85–1.09)	
Quartile 4 (very urban) (*n* = 1690)	411 (24.3)	0.95 (0.83–1.10)		0.98 (0.86–1.12)	

*Highest education level *					
None/up to p4 (*n* = 2112)	596 (28.2)	1	**<0.001**	1	**<0.001**
P5–P7 (*n* = 3365)	880 (26.2)	0.85 (0.77–0.95)		0.81 (0.73–0.91)	
Secondary+ (*n* = 2030)	431 (21.2)	0.66 (0.58–0.76)		0.64 (0.55–0.74)	

*Cigarette consumption*					
Never smokers (*n* = 6747)	1718 (25.5)	1	0.992	1	0.471
Ever smokers (*n* = 760)	189 (24.9)	1.00 (0.86–1.16)		0.94 (0.79–1.11)	

*Alcohol consumption*					
Never drinkers (*n* = 4844)	1276 (26.3)	1	**<0.001**	1	**<0.001**
Ex-drinkers (*n* = 583)	155 (26.6)	1.06 (0.90–1.25)		1.00 (0.84–1.20)	
Current drinkers (*n* = 2080)	476 (22.9)	0.81 (0.73–0.89)		0.78 (0.69–0.88)	

*HIV/ART status*					
Negative (*n* = 6821)	1711 (25.1)	1	**<0.001**	1	**<0.001**
Positive, on ART (*n* = 201)	33 (16.4)	0.54 (0.39–0.73)		0.57 (0.41–0.78)	
Positive, not on ART (*n* = 396)	133 (33.6)	1.62 (1.34–1.96)		1.76 (1.45–2.13)	

*BMI category*					
Underweight (*n* = 1701)	459 (27.0)	1	**<0.001**	1	**<0.001**
Normal (*n* = 4878)	1177 (24.1)	0.85 (0.76–0.95)		0.98 (0.87–1.10)	
Overweight (*n* = 730)	197 (27.0)	1.07 (0.91–1.27)		1.29 (1.07–1.55)	
Obese (*n* = 198)	74 (37.4)	1.70 (1.29–2.24)		2.08 (1.55–2.79)	

*Waist-hip ratio*					
Normal, ≤0.95-m, ≤0.80-f (*n* = 4440)	1065 (24.0)	1	**0.001**	1	**<0.001**
Abnormal, >0.95-m, >0.80-f (*n* = 3067)	842 (27.5)	1.17 (1.07–1.28)		1.55 (1.36–1.77)	

*Total cholesterol (mmol/L)*					
Desirable (≤5.2) (*n* = 7084)	1814 (25.6)	1	0.291	1	0.359
High (>5.2) (*n* = 423)	93 (22.0)	0.90 (0.74–1.09)		0.91 (0.74–1.12)	

*HbA1-C*					
Normal ≤6% (*n* = 7469)	1891 (25.3)	1	**0.004**	1	**0.014**
Raised ≥6.5% (*n* = 19)	11 (57.9)	3.65 (1.49–8.93)		3.17 (1.27–7.96)	

^*ϕ*^High atherogenic risk versus intermediate and low risk.
